# Inhibition of the Liver Enriched Protein FOXA2 Recovers HNF6 Activity in Human Colon Carcinoma and Liver Hepatoma Cells

**DOI:** 10.1371/journal.pone.0013344

**Published:** 2010-10-13

**Authors:** Frank Lehner, Ulf Kulik, Juergen Klempnauer, Juergen Borlak

**Affiliations:** 1 Department of General, Visceral and Transplantation Surgery, Hannover Medical School, Hannover, Germany; 2 Molecular Medicine and Medical Biotechnology, Fraunhofer Institute of Toxicology and Experimental Medicine, Hannover, Germany; 3 Center of Pharmacology and Toxicology, Hannover Medical School, Hannover, Germany; Chinese University of Hong Kong, Hong Kong

## Abstract

Recently, we demonstrated that the transcription factors HNF6 and FOXA2 function as key regulators in human colorectal liver metastases. To better understand their proposed inhibitory crosstalk, the consequences of functional knockdown of FOXA2 on HNF6 and C/EBPα activity were investigated in the human colon Caco-2 and HepG2 carcinoma cell lines. Specifically, siRNA-mediated gene silencing of FOXA2 repressed transcript expression by >80%. This resulted in a statistically significant 6-, 3-, 4-, and 8-fold increase in mRNA expression of HNF6 and of genes targeted by this transcription factor, e.g., HSP105B, CYP51, and C/EBPα, as determined by qRT-PCR. Thus, functional knockdown of FOXA2 recovered HNF6 activity. Furthermore, with nuclear extracts of Caco-2 cells no HNF6 DNA binding was observed, but expression of HNF1α, FOXA2, FOXA3, and HNF4α protein was abundant. We therefore transfected a plasmid encoding HNF6 into Caco-2 cells but also employed a retroviral vector to transfect HNF6 into HepG2 cells. This resulted in HNF6 protein expression with DNA binding activity being recovered as determined by EMSA band shift assays. Furthermore, by flow cytometry the consequences of HNF6 expression on cell cycle regulation in transfected cells was studied. Essentially, HNF6 inhibited cell cycle progression in the G2/M and G1 phase in Caco-2 and HepG2 cell lines, respectively. Here, proliferation was reduced by 80% and 50% in Caco-2 and HepG2 cells, respectively, as determined by the BrdU labeling assay. Therefore functional knockdown of FOXA2 recovered HNF6 activity and inhibited growth of tumor-cells and may possibly represent a novel therapeutic target in primary and secondary liver malignancies.

## Introduction

Colorectal cancer is the second leading cause of cancer death in the world. Nearly 800,000 new cases are diagnosed each year, and approximately 500,000 deaths have been estimated annually for the US alone [1], [2]. As of today, the molecular basis of metastatic spread of colonic tumor cells into the liver is unknown. There is need to improve an understanding of disease causing mechanisms as to develop novel and improved treatment opportunities.

Recently, we reported the regulation of some major hepatic nuclear factors in primary human colon cancer and colorectal liver metastases [3]. We found HNF6 expression to be absent in healthy colon or primary colon cancer, but observed abundant expression of unacetylated HNF6 in nuclear extracts of colorectal liver metastases. However, unacteylated HNF6 was unable to bind to targeted DNA sequences and to activate genes regulated by this factor. Because of its known interaction with HNF6 expression of FOXA2 was investigated, which we found to be highly upregulated in colorectal liver metastases. There is also evidence for HNF6 to serve as a coactivator protein thereby enhancing FOXA2 transcription, but FOXA2 represses HNF6 transcription and genes targeted by this transcription factor.

Based on our initial results and findings reported by others we wished to probe for the role of FOXA2 in the regulation of HNF6 activity in colorectal liver metastases [4], [5], [6]. We therefore studied the consequences of functional knockdown of FOXA2 on HNF6 DNA binding activity in the human colon cancer cell line Caco-2. We also investigated the role of HNF6 on cell cycle regulation in HepG2 cells, as this human hepatoma cell line is also devoid of the HNF6 protein. Indeed, HNF6 may function as a master regulatory protein in primary and secondary liver malignancies. Overall, our study aimed for an improved understanding of an inhibitory crosstalk between FOXA2 and HNF6 in metastasizing colon cancer and to utilize this knowledge for the development of an siRNA mediated therapeutic approach.

## Materials and Methods

### Cell culture

Caco-2 cells and HepG2 cells were obtained from the European Collection of Cell Cultures (ECACC, Salisbury, UK) and were cultured using conditions reported by Lampen et al. (Caco-2 cells) and Wilkening et al. (HepG2 cells) [7], [8].

### RNA isolation and cDNA synthesis

RNA was isolated with the RNeasy Mini Kit (Quiagen) according to the manufacturer's recommendation while cDNA synthesis was carried out as reported [3].

### Quantitative PCR analysis with the Roche Light Cycler System

Real time PCR was done with the LightCycler® according to the manufacture's recommendation (Roche Diagnostics, Penzberg, Germany) with oligonucleoitides previously reported [3]. SYBR® Green I was used as a fluorescent dye to determine the amplified PCR product after each cycle. The length of PCR products was checked by gel electrophoresis. Gene expression of FOXA2, C/EBPalpha, CYP51, HSP105B and HNF6 was determined with primers reported in [3] in a standard PCR reaction containing 50 ng of DNA, 4.0 mM MgCl2 and 2 µl of LightCycler DNA Master hybridisation mixture (LightCycler DNA Master Hybridization Probes, Roche Diagnostics Inc) in a total volume of 20 µl. The reaction was started with a denaturation step at 95°C for 20 seconds and amplification was performed for 50 cycles denaturation (95°C for 0 seconds; ramp rate 20°C per second), annealing (58°C for 8 seconds, ramp rate 20°C per second) and extension (72°C for 18 seconds, ramp rate 20°C per second). In the case of the mitochondrial ATPase the reaction was started with a denaturation step at 95° C for 20 seconds and amplification was performed for 50 cycles of denaturation (95°C for 0 seconds; ramp rate 20°C per second), annealing (55°C for 8 seconds, ramp rate 20°C per second) and extension (72°C for 18 seconds, ramp rate 20°C per second). PCR products were identified by monitoring DNA melting curves in the glas capillary. At the end of each extension phase fluorescence was observed and used for quantitative measurements within the linear range of amplification yielding calculated concentrations as relative units. Exact quantification was achieved by serial dilution with cDNA produced from total RNA extracts using serial dilution steps. The obtained values were divided by those of mitochrondrial ATPase to obtain expression values relative to the housekeeping gene.

### Isolation of nuclear extracts

Nuclear extracts from Caco-2 cells were isolated by the modified method of Dignam et al. [9]. Eleven days after seeding cells were washed twice with ice-cold PBS, scraped into microcentrifuge tubes and centrifuged for 5 min at 2000× g, 4°C. Cell pellets were resuspended in lysis buffer (10 mM Tris pH 7.4, 2 mM MgCl_2_, 140 mM NaCl, 1 mM DTT, 4 mM Pefabloc, 1% Aprotinin, 40 mM β-glycerophosphate, 1 mM sodiumorthovanadate and 0.5% TX100) at 4°C for 10 min (300 µl for 1×107 cells), transferred onto one volume of 50% sucrose in lysis buffer and centrifuged at 14000× g and 4°C for 10 min. Nuclei were resuspended in Dignam C buffer (20 mM Hepes pH 7.9, 25% glycerol, 420 mM NaCl, 1.5 mM MgCl_2_, 0.2 mM EDTA, 1 mM DTT, 4 mM Pefabloc, 1% Aprotinin, 40 mM β-glycerophosphate, 1 mM sodiumorthovanadate, 30 µl for 1×107 cells) and gently shaked at 4°C for 30 min. Nuclear debris was removed by centrifugation at 14000× g at 4°C for 10 min. Protein concentrations were determined according to the method of Smith et al. [10]. The extracts were aliquoted and stored at −70°C.

### Whole Tissue Extracts

Tissues were frozen in liquid nitrogen immediately after explantation and stored at −80°C until further analysis. Prior to protein extraction, about 150 mg of each frozen tissue were ground using pre-chilled mortar and pestle. Then the tissue was sonicated (UP 200 s, Dr. Hielscher) in 0.5 ml lysis buffer (5 mol/l urea, 2 mol/l thiourea, 40 mmol/l Tris, 4% CHAPS, 100 mmol/l DTT, 0.5% BioLyte 3–10; Biorad) on ice. After centrifugation at 12000 rpm the supernatant was recovered and the remaining pellet was resuspended in 0.5 ml lysis buffer (as described above). Extracts were combined and stored in aliquots at−80°C until analysis. Total protein concentrations of extracts were determined with the HCL-modified Bradford protein assay (Bio-Rad Protein Assay Dye Reagent Concentrate, Biorad).

### Western blotting experiments

Western immunoblotting was done as follows: Nuclear protein (30 µg) extracts of Caco-2 cell cultures were denaturated at 95°C for 5 min, followed by sodium dodecyl sulphate polyacrylamide gel electrophoresis (SDS-PAGE) on 12% polyacrylamide gels, and blotted onto a polyvinylidene difluoride membrane (NEN, Dreieich, Germany) at 350 mA for 2 h in a buffer containing 400 mM glycine and 50 mM Tris (pH 8.3). Non-specific binding sites were blocked with Rotiblock (Roth, Germany) in 1x TBS buffer. After electroblotting of proteins, membranes were incubated with polyclonal antibodies for HNF1 alpha (Santa Cruz sc6548), FOXA2 (Santa Cruz sc6554), FOXA3 (Santa Cruz sc5360), HNF4 alpha (Santa Cruz sc 6556), and HNF6 (kind gift of Dr. R. H. Costa, Chicago, Illinois, USA) for 1 h and washed 3 times with 1x TBS buffer containing 0.1% Tween-20 (Roth, Germany). Subsequently, the membranes were incubated with a 1∶5000 diluted anti-α rabbit antibody (Chemicon, Hofheim, Germany) for 1 h at room temperature, followed by 3 successive washes with 1x TBS buffer containing 0.1% Tween-20 (Roth, Germany). Immunoreactive proteins were visualized with a chemiluminescence reagent kit (NEN, Dreieich, Germany) according to the manufacturer's instructions, and bands were scanned with the Kodak Image Station CF 440 and analyzed using the Kodak 1D 3.5 imaging software (Eastman Kodak Company, USA).

### Design of EMSA-oligos for HNF6 binding sites in promotor sequences of human genes

Known binding sites of HNF6 (ONECUT1) were collected from the TRANSFAC database, (www.biobase.de). The motive search was done with TRANSFAC release 9.4. To retrieve promoters of human genes the TRANSPro release 2.1 was employed. In the case of HNF6 the position weight matrix (M00639; V$HNF6_Q6) was used that was originally constructed on the basis of 13 known binding sequences of HNF6 targeted promoters. The design for the oligo probes for HNF6 and FOXA2 was optimized as previously reported [3].

### Annealing of synthetic oligonucleotides and [32P] labeling

Oligonucleotides were designed enharboring high affinity HNF6 consensus site in gene specific promoters. Oligonucleotides were annealed at a concentration of 19.2 pM · µL^−1^ in 200 mM Tris (pH 7.6), 100 mM MgCl_2_ and 500 mM NaCl at 80°C for 10 min, then cooled slowly to room temperature overnight and stored at 4°C. Annealed oligonucleotides were diluted to 1∶10 in Tris-EDTA buffer (1 mM EDTA, 10 mM Tris, pH 8.0) and labelled using [32P] ATP (Amersham Biosciences Europe GmbH, Freiburg, Germany, 250 µCi, 3,000 Ci · mM^−1^) and T4 polynucleotide kinase (New England Biolabs GmbH, Frankfurt am Main, Germany). End-labelled probes were separated from unincorporated [32P] ATP with a Microspin G-25 Column (Amersham Biosciences Europe GmbH, Freiburg, Germany) and eluted in a final volume of 100 µL.

### Electrophoretic mobility band shift assay (EMSA)

EMSA assays were carried out as described in [11]. Briefly, 5 µg of CaCo2 nuclear extract were incubated with the binding buffer consisting of 25 mM HEPES (pH 7.6), 5 mM MgCl_2_, 34 mM KCl, 2 mM DTT, 2 mM Pefablock (Roche Diagnostics GmbH, Mannheim, Germany), 0.5 µL aprotinin (2.2 mg · mL^−1^, Sigma-Aldrich Chemie GmbH, Taufkirchen, Germany), 50 ng poly (dl-dC) and 80 ng bovine serum albumin (PAA Laboratories GmbH, Cölbe, Germany). The binding reaction was carried out for 20 min on ice, and free DNA and DNA-protein complexes were resolved on a 6% polyacrylamide gel. Furthermore, a specific HNF6 and/or HNF4α antibody (Santa Cruz Biotechnology Inc., Heidelberg, Germany) was added to the reaction mix 10 min before addition of the labelled probe. In the case of NGN3, no commercial antibody is available. Thus, a competition assay at x100 and x500-fold access of unlabeled oligonucelotide probe specific for NGN3 was used. Gels were blotted to Whatman 3 MM paper, dried under vacuum, exposed to imaging screens (Imaging Screen-K, Bio-Rad Laboratories GmbH, Munich, Germany) for autoradiography overnight at room temperature and analyzed using a phosphor imaging system (Molecular Imager FX pro plus; Bio-Rad Laboratories GmbH, Munich, Germany) and the Quantity One Version 4.2.2 software (Bio-Rad Laboratories GmbH, Munich, Germany).

### Viral expression system for HNF6

Based on a method of Soneoka et al. a three-plasmid expression system was used for the production of retroviral vetors [12]. Specifically, this murine retroviral system employs the Vesicular Stomatitis Virus G protein (VSV-G wt) in the packaging of viral particles. Note, VSV-G protein mediates the viral entry via a ubiquitously expressed receptor on target cell surface. The viral particle production is performed in HEK293T cells with a protocol based on the transient packaging method established by Soneoka et al. [12]. In this system genes encoding the structural proteins, the envelope protein and the gene of interest are expressed by different plasmids. The three-plasmids are transfected simultaneously into the packing HEK293T cells. The viral particles contain only parts of the parental organism including the 5′LTR, 3′LTR and the packing signal Psi. Since genes encoding the structural proteins are not transfected with the viral particle, the produced viruses are infectious but replication-incompetent. The viral particles permitted transfer of the HNF6 coding sequence to HepG2 cells.

### Transfection of HNF6 into Caco-2 cells and the HepG2 human hepatoma cell line

To confirm the proposed inhibitory cross-talk of FOXA2 and HNF6 and to determine its effect on gene expression and cell cycle regulation the colon carcinoma cell line Caco-2 cells and the human hepatoma cell line HepG2 was transfected with a HNF6 containing plasmid. The plasmid was the kind gift of Dr. Lémaigre, Université catholique de Louvain, Belgium. Importantly, healthy colonic epithelium and colonic cancers do not express HNF6 but expression of unacetylated HNF6 was observed in nuclear extracts of colorectal liver metastases [3]. Therefore, it became necessary to express HNF6 in target cell lines to enable mechanistic studies. To be able to investigate the inhibitory cross talk of the FOXA2 and HNF6 transcription factor proteins Caco-2 cells were transfected with an HNF6 plasmid and Lipofectamine 2000 (Invitrogen), that is a cationic-lipid transfection reagent according to the manufacture's recommendation. In the case of HepG2 cells a retroviral transfection plasmid was used to improve the level of HNF6 expression in HepG2 cells. Note, the expression of HNF6 by use of the retroviral transfection plasmids was similar to healthy liver (data not shown) when the protocol of Soneoka et al. was used as described above [12].

Briefly, two vectors were received from the laboratory of Dr. Lémaigre to enable efficient transfection of HNF6 (Hormone and Metabolic Research Unit, Institute of Cellular Pathology, Université catholique de Louvain, Brussels, Belgium). HNF6 was cloned into (a) V-831, pCMV-MCS (Stratagene), a mammalian expression vector containing a multiple cloning site (MCS), a CMV promoter, and other elements for high-level gene expression; (b) V-894, pIRES2-EGFP (Clontech, discontinued), which contains an internal ribosome entry site (RES; 1,2) of the encephalomyocarditis virus (ECMV) between the MCS and the enhanced green fluorescent protein (eGFP) coding region. This permitted both the gene of interest (cloned into the MCS) and the eGFP gene to be translated from a single bicistronic mRNA. Plasmid DNA was isolated using the Maxiprep endotoxin-free kit (Qiagen). The DNA was precipitated with EtOH at 4000 rpm for 90 min and 2x washings at 4000 rpm for 60 min. The HNF6 insert was confirmed by RLFP with *Bam*HI/*Eco*RI for V-894 and *Bam*HI/*Xho*I for V-831. The product was approximately 1.6 kb after restriction enzyme digestion.

Cells (200,000 per 35-mm well) were seeded in a six-well culture plate containing 2 ml per well of Dulbeco's modified Eagles medium (DMEM) with fetal calf serum (end conc. 8.7%), glutamine (end conc. 2x), and penicillin/streptomycin (end conc. 2 x). Cells were grown at 37°C in a humidified 5% CO_2_/approx. 95% air atmosphere. The medium was prepared as follows: 500 ml DMEM +50 ml of 10% FCS +12 ml of 100x L-glutamine and 12 ml of 100x penicillin/streptomycin.

**Figure 1:**
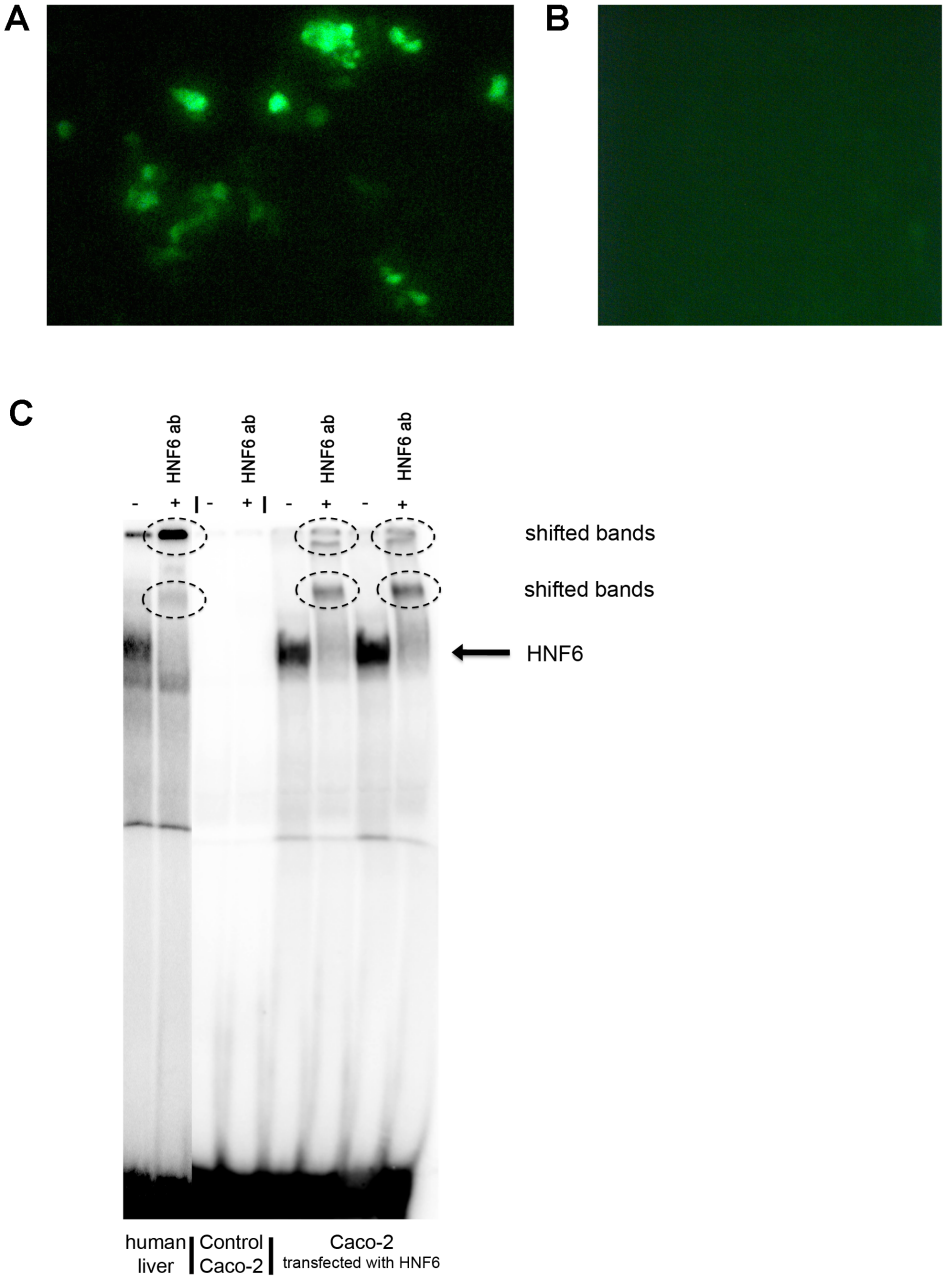
HNF6 protein expression and DNA binding activity in the human colon carcinoma cell line Caco-2. A. Image of fluorescent microscopy of HNF6 plasmid-transfected into Caco-2 cells, magnification 20x; B. empty vector control; C. electromobility band shift assay (EMSA) demonstrating HNF6 DNA binding activity with nuclear extracts isolated from healthy human liver (positive control), empty vector control and HNF6-transfected Caco-2 cells; addition of an antibody specific for HNF6 shifted bands as denoted by the circle.

Prior to transfection (1 h), DMEM medium was removed and cells were washed with PBS. The medium was then replaced with Opti-MEM I medium, a versatile chemically defined medium formulated to significantly reduce the amount of serum required for cultivating mammalian cells *in vitro*. It is a modification of Eagle's Minimal Essential Medium, buffered with HEPES and sodium bicarbonate, and supplemented with hypoxanthine, thymidine, sodium pyruvate, L-glutamine or GlutaMAX, trace elements, and growth factors. The protein level is minimal (15 mg/ml), with insulin and transferrin being the only protein supplements. Phenol red is included at a reduced concentration as a pH indicator. Transfection was carried out using Lipofectamine 2000 (Invitrogen), a cationic-lipid transfection reagent according to the manufacture's recommendation. Efficiency of transfection was assed qualitatively by fluorescent microscopy of which an example is given in Fig. 1a.

In the case of HepG2, the HNF6 containing plasmid (see above) was cloned into the pczCFG5.1MCS vector, which is one necessary component of the three-vector transduction system. In order to produce replication-deficient retroviral particles genes encoding the structural and envelope proteins, as well as the HNF6 coding plasmid were transfect into the human embryonic kidney 293T packaging cells. Then, the viral particles derived from the HEK293T cell line were harvested and used to transfect HNF6 into HepG2 cells as detailed below.

### Protocol for packaging of retroviral vectors

Packaging of retroviral particles was carried out with HEK293T cells at a density of 4×10^6^ cells per 10 cm dish. At day 1 usually the confluency of HEK293T cells was between 60–80%. The transfection of the HEK293T cells with the retroviral vectors was achieved using the PEI protocol as originally described by Soneoka et al. [12]. Here the plasmid pHIT60 encodes gag-pol whereas the plasmid pcz-VSV-Gwt encodes the envelope protein from Vesicular Stomatitis Virus while plasmid pcz-CFG2-HNF6 (see above) contains the HNF6 coding sequence. The vectors without HNF6 were the kind gift of Dr. Achim Renne, Department of Neurological Surgery, University of Dresden, Germany.

To induce activity of the CMV promotor the transfection medium was replaced with medium containing sodium butyrate at a final concentration of 10 mM. Note, polybrene enhances viral adherence on cells. The medium from the HEK293T cells was collected and filtered trough 0.2 µm filters. Polybrene was added at a concentration of 8 µg/ml and the HepG2 cells were incubated with this medium over night to induce HNF6 expression.

### Small-interference FOXA2 RNA (siRNA)-mediated knockdown in Caco-2

**Figure 2:**
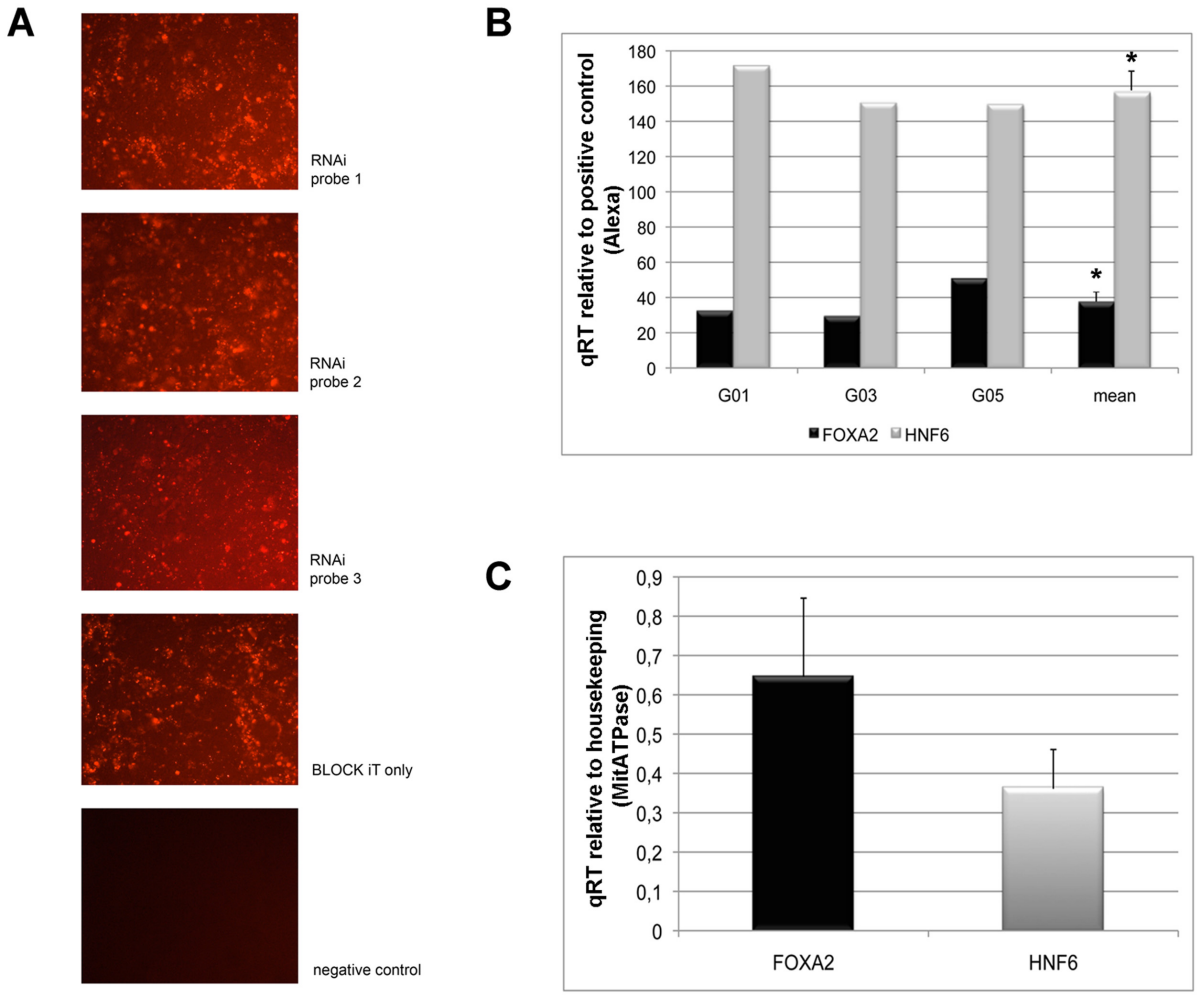
Functional knockdown of FOXA2 in the human colon carcinoma cell line Caco-2. A. Images of fluorescent microscopy of Caco-2 cells transfected with different FOXA2 siRNA probes and empty vector control. Note, the transfection efficiency was similar amongst the three different siRNA probes while functional knock down of FOXA2 was best with one probe termed FOXA2HSS142473 (details are given by the manufacture, Invitrogen, Paisley, Great Britain); G01, G03 and G05 refer to three independent individual experiments; B. FOXA2 and HNF6 gene expression after functional knockdown of FOXA2 in three independent experiments, *p<0.05 when compared to untreated Caco-2 cells; C. Gene expression of HNF6 and FOXA2 in untreated Caco-2 cells of n = 3 independent experiments, *p<0.05. HNF6 gene expression is significantly less when compared to FOXA2 gene expression.

Caco-2 cells were cultured to 70–80% of confluence and were transfected with 3 different FOXA2 siRNA probes (see FOXA2 Stealth^™^ (Invitrogen) as originally designed by Invitrogen. These probes were used according to the manufacture's recommendations and allowed verification of phenotypic changes as well as control of off-target effects. Transfection efficiency was controlled by the Block-iT™ Alexa Fluor® Red Fluorescent Oligo (Invitrogen). This red-labeled dsRNA oligomer is designed for use in RNAi experiments to facilitate assessment and optimization of dsRNA oligonucleotides delivery into mammalian cells by use of cationic lipids (Lipofectamine 2000). In Fig. 2 images of individual FOXA2 siRNA probes transfections and of BLOCK-IT Alexa Fluor Red Fluorescent Oligo as well as a negative control are depicted. Notably, Caco-2 cells were incubated with various FOXA2 siRNA oligonucleotide for 48 h to down regulate FOXA2 gene expression. FITC-labeled scrambled siRNA (Control-FITC block-it fluorescent Oligo #2013, Invitrogen, Germany) was used as a negative transfection control.

### Measurement of RNAi activity

Quantitative RT-PCR was applied to determine the gene expression of FOXA2 after siRNA knock-down and of HNF6 and genes regulated by this factor, i.e. C/EBPalpha, HSP105B and CYP51 using the oligonucleotide probes and the protocol described above.

### Cell cycle and cell proliferation assay

Cells were plated in 96-well microtiter plates at a density of 5000 cells/well 24 h prior to treatment. Cell cycle and cell proliferation were measured using the CycleTest Plus Reagent and the BrdU labeling kit according to the manufacture's recommendations (see below).

### Cell cycle analysis

The effects of HNF6 expression on the cell cycle were studied by flow cytometry. Cells were plated in six-well sterile plastic plates at a density of 10^5^ −2×10^5^ cells/well and were allowed to attach for 24 h. Then cells were collected by trypsinization and DNA staining was done with the CellTest Plus Reagent Kit (Becton Dickinson Immunocytometry Systems, San Jose, California, USA). According to the manufacturer's instruction cells were washed with a buffer solution containing sodium citrate, sucrose, and dimethyl sulfoxide (DMSO). Then cells were incubated according to a three-step sequence: a) 10 min at room temperature with solution A containing trypsin in a spermine tetrahydrochloride detergent buffer (to digest cell membranes and cytoskeleton); b) 10 min at room temperature with solution B containing a trypsin inhibitor and ribonuclease A in citrate-stabilizing buffer with spermine terahydrochloride (to inhibit the trypsin activity and to digest RNA); c) 15 min in the refrigerator with solution C containing propidium iodide and spermine tetrahydrochloride in citrate-stabilizing buffer. Analysis was performed using a FACScan (Becton Dickinson GmbH Immunozytometrische Systeme, Heidelberg, Germany), and data analysis was carried out with CELLQuest software, while cell cycle distribution was determined using the Modifit software (Verity Software House, Inc.).

### BrdU cell proliferation assay

BrdU incorporation was measured using the BrdU Cell Proliferation Assay (Merck, Darmstadt, Germany) according to the manufacturer's instructions. Cells were labeled with BrdU (1∶100) for the last 4 h of incubation. Cells were washed, fixated, and incubated with mouse anti-BrdU antibody (1∶100; 100 µl/well) for 1 h at room temperature. Antibody labeling was detected by secondary peroxidase-coupled goat-anti-mouse antibody (1∶1000, 100 µl/well; 30 min at room temperature). After washing, peroxidase substrate was added for 15 min. The peroxidase reaction was stopped by adding 100 ml 2.5N sulfuric acid, and absorbance was measured using dual wavelengths of 450 and 595 nm.

### Statistical analysis

The Wilcoxon signed rank test and the student's t-test was used to determine significance with *P*<0.05 being statistically significant.

## Results

### Recovery of HNF6 expression in Caco-2 cell cultures

Initially, studies were carried out with the human colon adenocarcinoma cell line Caco-2. Unlike colorectal liver metastases which do express HNF6, primary colonic cancer and Caco-2 cells do not express detectable levels of this protein. We therefore employed an HNF6-containing plasmid and studied DNA binding activity of the coded protein upon transfection. The choice of vector permitted imaging of HNF6 (cloned into the MCS) by fluorescence microscopy. As shown in Fig. 1A Caco-2 cells were successfully transfect with HNF6 but the transfection efficacy varied amongst individual experiments, while Fig. 1B exemplifies empty vector transfections. To further probe for HNF6 binding activity we performed EMSA band shift assays. As depicted in Fig. 1C and unlike controls we observed HNF6 nuclear protein expression and DNA binding activity in transfected Caco-2cells. Here healthy human liver nuclear protein extracts served as control. Notably, expression level of HNF6 protein transfected into Caco-2 cells was similar to that of human liver. Addition of an antibody that specifically recognizes HNF6 completely removed the protein bound to an HNF6 optimized oligonucleotide probe therefore confirming specificity of the assay. With nuclear extracts of HNF6 transfected Caco-2 cells additional bands are visible suggesting protein-protein interactions at an HNF6 optimized oligonucleotide probe.

### siRNA-mediated functional knockdown of FOXA2

Evidence from our own laboratory and other investigators suggested a regulatory loop for FOXA2 with HNF6 [3], [13]. Indeed, HNF6 functions as a coactivator protein to potentiate the transcriptional activity of FOXA2 [13]. Furthermore, it was shown earlier that a C/EBPα-HNF6 protein complex stimulates HNF6 and FOXA2 transcriptional activity through recruitment of the CBP coactivator protein [14].

**Figure 3:**
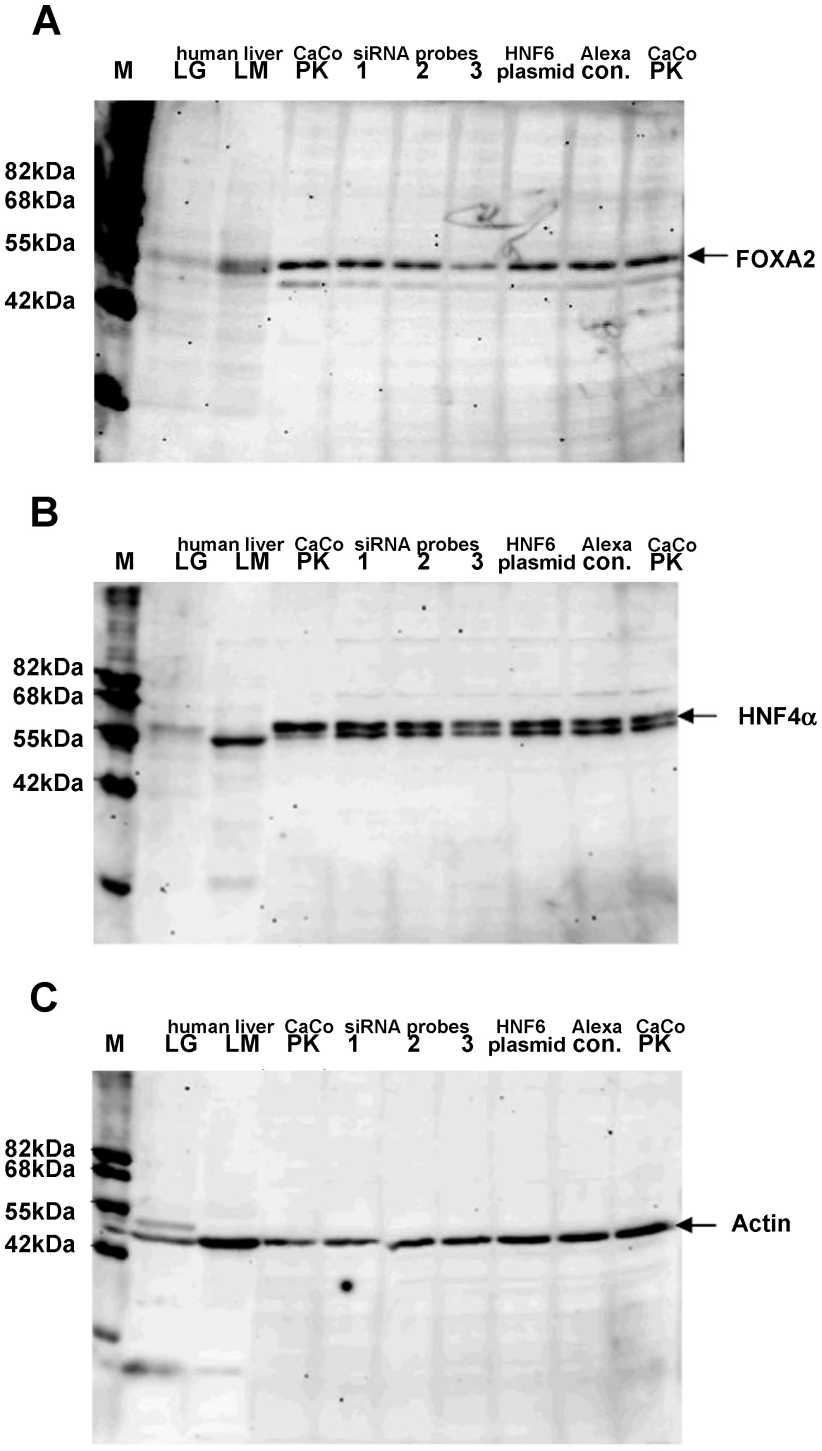
Western blotting of FOXA2 (A), HNF4α (B) and Actin (C) in CaCo-2 cells and human liver. Note, the HNF4α served as a positive control while Actin was used as loading control. Human liver nuclear extracts served as additional positive control. One of the siRNA oligonucleotides reduced FOXA2 protein significantly (see siRNA probe 3). This probe also affected expression of HNF4α while HNF6 plasmid expression had no effect on FOXA2 or HNF4α protein expression. M = molecular weight standard; LG = nuclear extracts of healthy liver; LM = nuclear extracts of colorectal liver metastases; PK = nuclear extracts of untreated Caco-2 cells; lanes 1-3 refers to different siRNA probes.

To probe for an inhibitory FOXA2-HNF6 crosstalk a small-interference RNA-mediated knockdown of FOXA2 in Caco-2 cells was carried out at a confluency of about 70%. In Fig. 2A images of transfections with different siRNA probes and the empty vector control are depicted. Essentially the transfection efficiency of the different RNAi probes was similar as was that of the BLOCK-IT “empty vector control” which was used to monitor the efficiency of transfection. Based on qRT-PCR a statistically significant nearly 80% knockdown efficiency of FOXA2 gene expression was achieved. This resulted in reduced FOXA2 protein expression as illustrated in Fig. 3 albeit at different levels, when individual siRNA probes were compared. Indeed, a total of 6 individual experiments were carried out. In 4 out of 6 experiments the data were robust and reliable suggesting that only some probes are efficient in silencing FOXA2 gene expression. Fig. 2B depicts the results of FOXA2 and HNF6 gene expression of at least n = 3 independent siRNA experiments. Notably, functional knockdown of FOXA2 resulted in a significant 4-fold increase in HNF6 gene expression. For comparison the expression of FOXA2 and HNF6 in untreated CaCo-2 cells is depicted in Fig. 2 C. With Caco-2 cells FOXA2 gene expression was nearly twice that of HNF6 (see Fig. 2C). Note, in Caco-2 cells HNF6 transcripts were not translated into protein.

**Figure 4:**
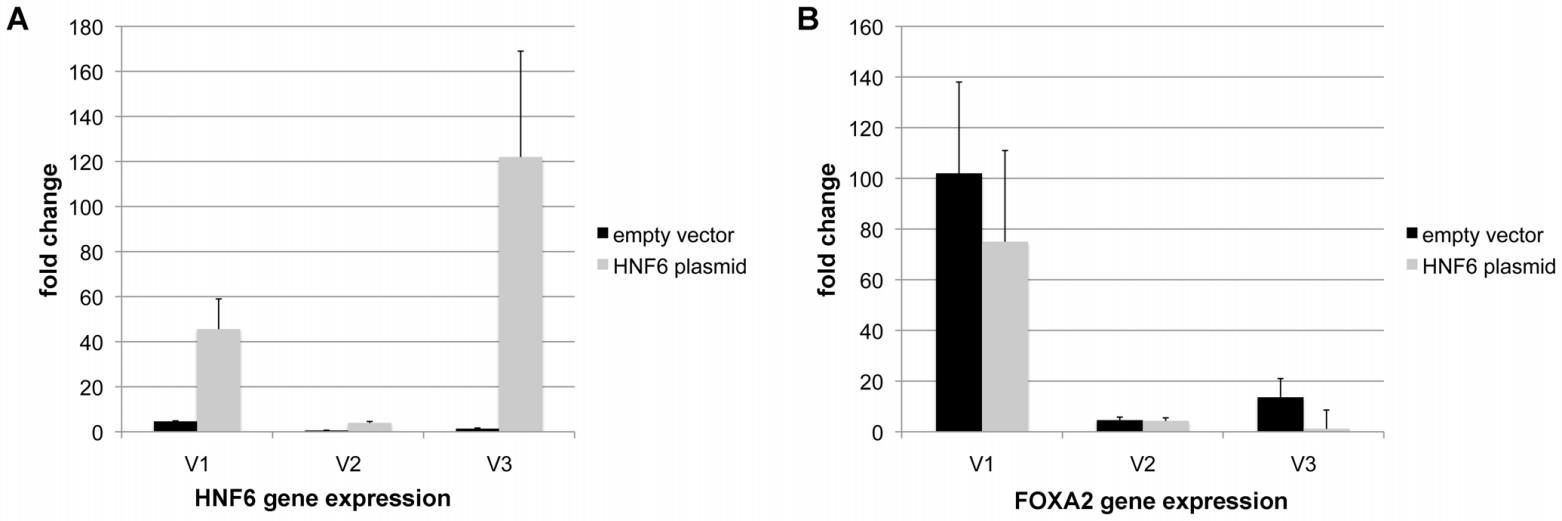
qRT-PCR of HNF6 and FOXA2 gene expression in HNF6 plasmid expressing CaCo-2 cells. Shown are n = 3 independent experiments (denoted by V1 to V3) where HNF6 plasmid expression resulted in significant increase in HNF6 mRNA transcripts (see panel A). The efficiency of HNF6 transfection varied considerably. In HNF6 transfected Caco-2 cells expression of FOXA2 mRNA transcripts was investigated (see panel B). FOXA2 gene expression was reduced. Each bare represents the mean of at least n = 3 independent measurements.

By qRT-PCR the gene expression of HNF6 and FOXA2 was studied in cells transfected with the HNF6 plasmid. As shown in Fig. 4A plasmid gene expression of HNF6 varied amongst individual experiments and depended on the transfection efficiency but was up to 120-fold increased, as compared to the empty vector control. Note, efficient HNF6 plasmid expression resulted in a reduced FOXA2 gene expression but the effects differed amongst individual experiments (see. Fig. 4B).

**Figure 5:**
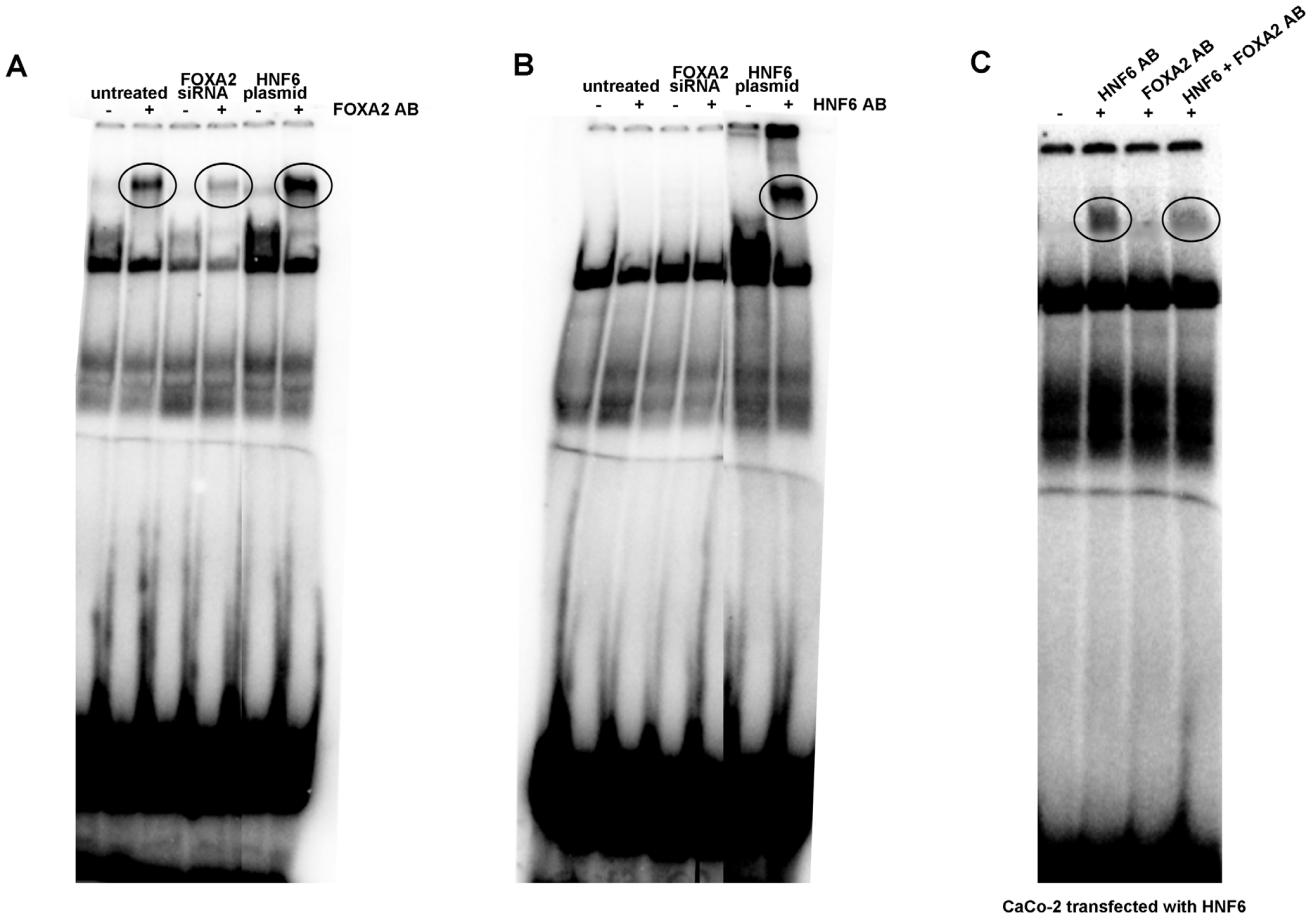
Electromobility band shift assay with nuclear extracts isolated from either untreated or FOXA2 siRNA or HNF6 plasmid expression cells. Panel A: Depicted is the DNA binding activity of FOXA2 to an optimized probe. Specificity of the DNA binding is confirmed by adding a FOXA2 antibody which resulted in a shifted band. A circle marks the shifted band. Note, siRNA of FOXA2 resulted in a marked reduced DNA binding of the FOXA2 protein to its cognate recognition site. Importantly, FOXA2 DNA binding activity is strongly increased in HNF6 plasmid expressing cells. Panel B: Depicted is the DNA binding activity of HNF6 to an optimized probe. Specificity of the DNA binding is confirmed by adding a HNF6 antibody, which resulted in a shifted band which is marked by a circle. In untreated cells no HNF6 DNA binding is observed and for such cell cultures FOXA2 siRNA did not influence HNF6 DNA binding activity. In HNF6 plasmid expressing cells high HNF6 DNA binding activity is observed. Panel C: Depicted is the DNA binding activity of HNF6 transfected Caco-2 cell nuclear extracts to an optimized probe. A marked reduction in DNA binding activity of HNF6 is observed when an antibody recognizing FOXA2 is added concomitantly. Likewise, addition of antibodies specific for HNF6 and FOXA2 reduced HNF6 DNA binding activity to an HNF6 optimized oligonucleotide probe.

Furthermore, the DNA binding of FOXA2 and HNF6 was investigated in CaCo-2 cells after functional knock down of FOXA2. As shown in Fig. 5A DNA binding activity of FOXA2 to an optimized FOXA2 oligonucleotide probe was significantly reduced after functional knock down of FOXA2, while in HNF6 plasmid expressing Caco-2 cells FOXA2 DNA binding was significantly increased (see panel A). As depicted in panel B the DNA binding of HNF6 (not FOXA2!) to an optimized HNF6 oligonucleotide probe was significantly increased in HNF6 plasmid expression CaCo-2 cells, whereas no DNA binding was observed in untreated or FOXA siRNA treated CaCo-2 cells. This is of no surprise, as CaCo-2 cells do not express HNF6 protein at detectable level. Notably, we recently demonstrated that epithelium of healthy human colon does not express HNF6 mRNA transcripts and protein expression levels [15]. In this study Lehner et al. investigated the gene expression patterning of HNF6 in different segments of the human intestine and demonstrated a significant local and segmental differences in the expression of HNF6 and other liver enriched transcription factors in the human intestine which impacts epithelial cell biology of the gut [15]. Consequently, FOXA2 siRNA does not recover HNF6 DNA binding activity in unmodified CaCo-2 cells as these cells do not express HNF6. However, the DNA binding activity of HNF6 in CaCo-2 cell HNF6 plasmid expressing cells was significantly reduced when a FOXA2 antibody was used in EMSA band shift assays with an HNF6 optimized oligonucleotide probe (see Fig. 5 panel C). Thus, the previously described inhibitory cross talk between HNF6 and FOXA2 can be recapitulated at the level of DNA binding activity in HNF6 plasmid expressing CaCo-2 cells. Overall, a protein-protein interaction amongst FOXA2 and HNF6 to an HNF6 optimized oligonucleotide probe is evidenced (see lane 4 of Fig. 5C).

Results from Western blotting experiments are depicted in Figure 3. Notably, FOXA2 siRNA Probe 3 caused a significant reduction in FOXA2 and a minor reduction in HNF4alpha expression while actin served as a loading control.

**Figure 6:**
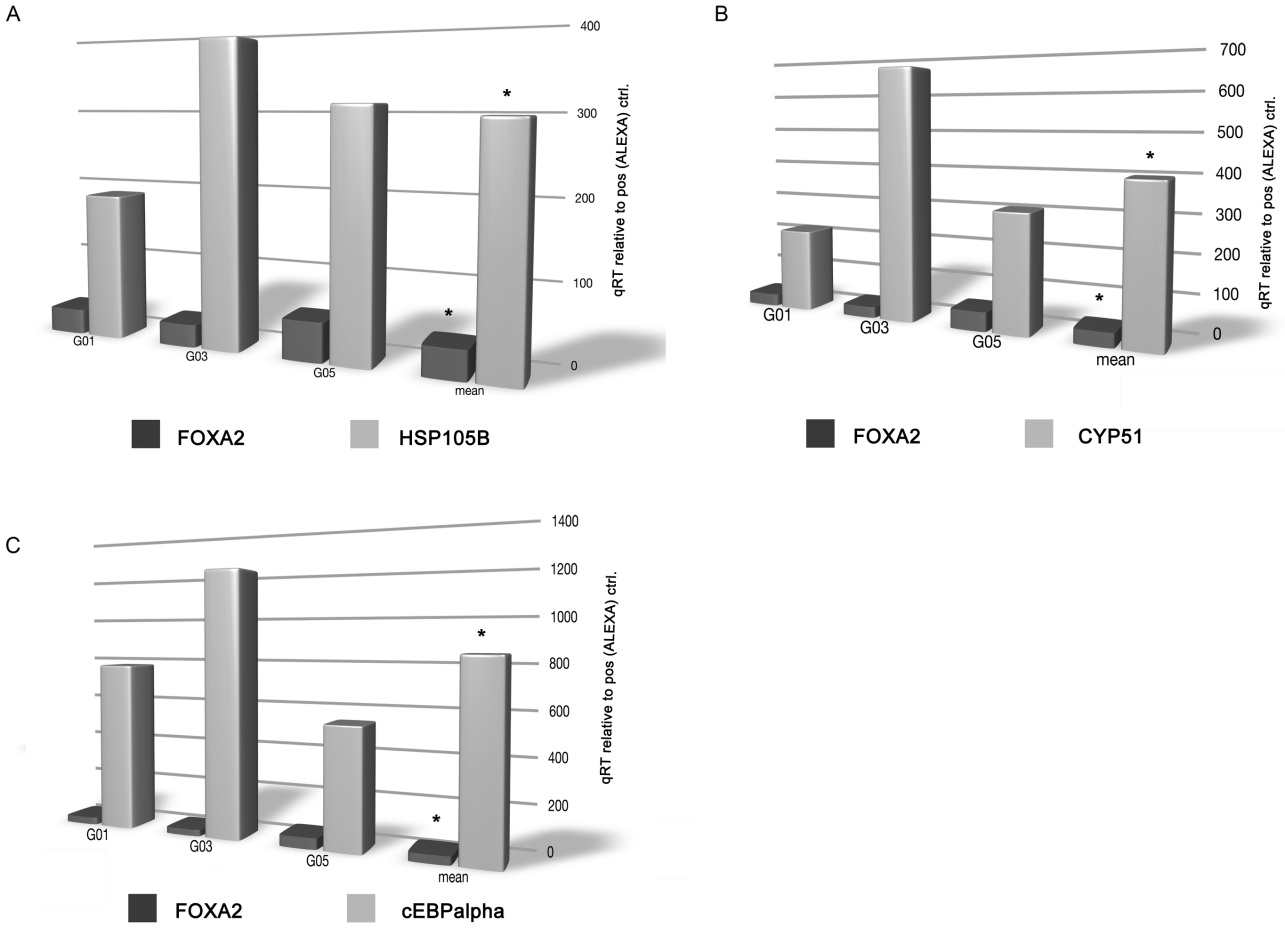
Gene expression of FOXA2 and of the HNF6 target genes HSP105B (see panel A), CYP51 (see panel B) and C/EBPα (see panel C) in the human carcinoma cell line Caco-2 after siRNA-mediated functional knockdown of FOXA2. Results represents the mean of n = 3 individual experiments; G01, G03 and G05 refer to individual experiments. *p<0.05 when compared to untreated CaCo-2 cells.

Based on the study of Odom et al. [16], who employed a CHIP-chip protocol to identify HNF6 target genes, three genes targeted by this factor were selected. As shown in Fig. 6 siRNA-mediated functional knockdown of FOXA2 resulted in a 3-fold, 4-fold, and 8-fold increase in gene expression of HSP105B, CYP51, and C/EBPα, respectively.

### Cell cycle and BrdU labeling experiments with the Caco-2 and HepG2 human cancer cell lines

**Figure 7:**
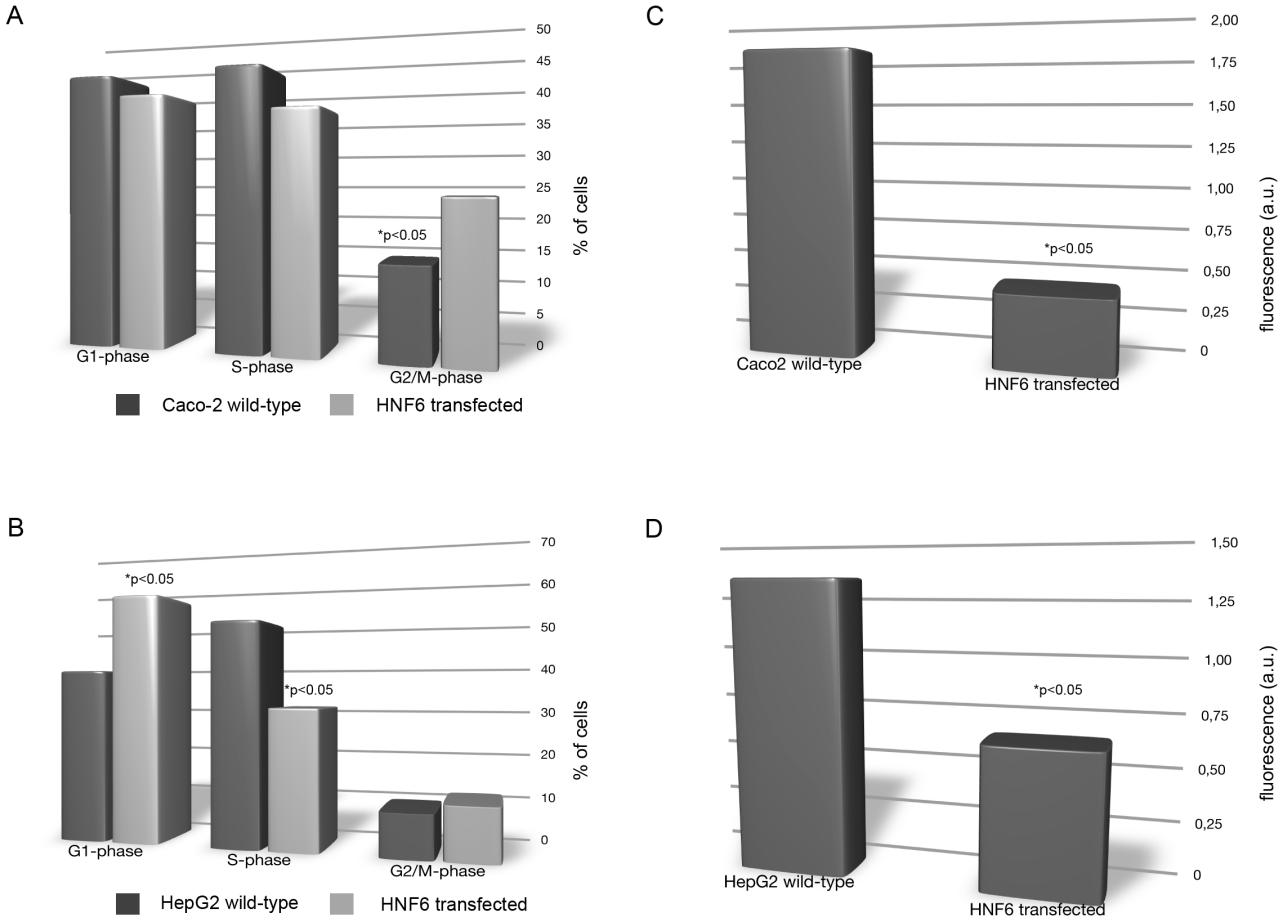
Cell cycle analysis and BrdU labeling in Caco-2 and HepG2 cells. A. Cell cycle analysis of Caco-2 and HNF6-transfected Caco-2 cells by flow cytometry. B. Cell cycle of HepG2 and HNF6-transfected HepG2 cells by flow cytometry. C. BrdU labeling of the human colon cancer cell line Caco-2 transfected with HNF6. P<0.05 when compared to untransfected cells. D. BrdU labeling of the human liver cancer cell line HepG2 transfected with HNF6. P<0.05 when compared to untransfected cells.

To delineate a possible role of HNF6 on cell division BrdU labeling and cell cycle analysis studies were carried out. As compared to the empty vector, HNF6 transfection caused a highly significant cell cycle arrest in the G2/M phase in Caco-2 cells (Fig. 7A) and in the G1 an S-phase of HepG2 cells, respectively (Fig.7B). Likewise, cell proliferation was significantly reduced by 80% and 50% in HNF6-transfected Caco-2 cells (Fig. 7C) and HepG2 cells (Fig. 7D).

## Discussion

Colorectal liver metastases is a major cause of cancer morbidity and this study aimed for an improved understanding of an inhibitory cross talk of FOXA2 and HNF6 in secondary liver malignancies. Specifically, the carcinoma cell lines Caco-2 and HepG2 enabled mechanistic investigations into FOXA2 on HNF6 nuclear protein activity. Because HNF6 is not expressed in healthy colonic epithelium and colonic cancers but in colorectal liver metastases it was necessary to employ an experimental strategy by which HNF6 is transfected into cancer cell lines as to permit mechanistic studies. Thus, HNF6 was transfected by different means to the cancer cell lines Caco-2 and HepG2 which are colon and human hepatoma cell lines. The transfected protein was stable and DNA binding activity of HNF6 could be evidenced by EMSA band shift assays. Notably, we used the same optimized oligonucleotide probes to investigate DNA binding of HNF6 as reported in our initial study on human colorectal liver metastases [3]. In this study no DNA binding of HNF6 was observed with extract of nuclear proteins isolated from colorectal metastatic liver tumors, even though abundant expression of the HNF6 protein was seen. In fact, HNF6 DNA binding was selectively abrogated through lack of posttranscriptional acetylation [3]. In the present study we evidenced transfection of the HNF6 protein in Caco-2 cell cultures to result in HNF6 DNA binding activity (see Fig. 1C). Note, no HNF6 DNA binding activity was observed with control Caco-2 cell cultures (see Fig. 1C). Because of the hypothesized inhibitory crosstalk between FOXA2 and HNF6 we investigated the consequences of functional knockdown of FOXA2 on HNF6 gene expression. As shown in Fig. 2B an approximately 4-fold increase in HNF6 gene expression was determined in FOXA2 siRNA-transfected Caco-2 cell cultures. Thus, FOXA2 knockdown recovered HNF6 activity.

Essentially, there are two seminal studies on the transcriptional activity of HNF6 and its association with the FOXA2 protein [17], [18]. There is evidence for HNF6 to serve as co-activator protein to enhance FOXA2 transcription, whereas FOXA2 protein represses transcriptional activation of HNF6 target genes through inhibition of HNF6 DNA binding activity. It was demonstrated that on the FOXA2 promoter the HNF6 and FOXA2 protein interaction stimulates recruitment of the p300/CBP histone acetyltransferase proteins, which interacts with the RNA polymerase II transcriptional machinery [18]. Conversely, binding of FOXA2 and its physical interaction with HNF6 inhibited HNF6 dependent transcriptional activation of targeted genes, as only acetylated HNF6 displayed DNA binding activity [18]. In the study of Poll et al. the regulation of the HNF6 promoter was studied in detail thereby delineating transcription factor binding sites [17]. Through DNase I footprinting experiments binding sites for the transcription factors Sp1 and STAT5 were identified in the HNF6 promoter. In addition, the investigators identified an intronic enhancer termed I3. Such footprint analysis defined transcription factor binding sites for C/EBP, FOXA, HNF6, NF1 and HNF1/PDX1 proteins. In subsequent luciferase gene driven HNF6 promoter activity studies it was demonstrated that mutation of FOXA and HNF1 binding sites in the intronic region I3 reduced transcriptional activity of the HNF6 gene. From these results it was concluded that FOXA and HNF1 proteins are critical regulators of HNF6 expression and that binding of FOXA2 modifies chromatin structure to permit access of other transcription factors, as detailed in the study of Cirillo et al. [19].

Taken collectively, FOXA2 likely controled expression of HNF6 through physical interaction with HNF6 that has been shown to bind to an intronic sequence I3 thereby controlling expression of genes at least in pancreatic precursor cells of the endoderm, as reported in detail by Poll et al. [17]. Nonetheless, there is controversy regarding an interaction between HNF6 and FOXA2 in the liver. Indeed, in the study of Rubins et al. hepatocyte specific gene ablation of FOXA2 was achieved. Here, HNF6 activitiy at targeted promoters in vivo appeared to be independent of the FOXA2 protein [20], while in the study of Rubins et al. it was also demonstrated that FOXA2 does synergize with HNF6 at some HNF6 targeted gene promoters to possibly facilitate activation of transcription. There is clear evidence for FOXA proteins to induce chromatin remodeling, thereby permitting access of other transcription factors to induce gene transcription. Possibly, the association between HNF6 and FOXA2 functions in a cell type specific manner and may be restricted to some targeted gene promoters. Likewise, in the study of Rausa et al. coimmunoprecipitation experiments demonstrated that the HNF6 Cut domain was sufficient to interact with the FOXA2 protein an that retention of the HNF6 Cut and Homeodomain was required for interaction with the p300/CBP histone acetyltransferase proteins to induce transcriptional synergy [18]. Notably, these studies were carried out in HepG2 cells as utilized in the present study where HNF6 was transfected into HepG2 and CaCo-2 cells.

We further demonstrated gene expression of HNF6-regulated genes, namely, HSP105B, CYP51, and C/EBPα to be significantly upregulated upon siRNA-mediated functional knockdown of FOXA2. Indeed, functional knockdown of FOXA2 induced transcriptional regulation of C/EBPα, a transcription factor that causes arrests of cell proliferation through direct inhibition of Cdk2 and Cdk4 [21]. In the study of Odom et al., who employed a CHIP-chip protocol to identify HNF6 target genes, it was demonstrated that C/EBPalpha is a bonafide target of this protein [16]. Furthermore, an association between HNF6 and C/EBPalpha had been reported by Yoshida et al. [14]. Here, a C/EBPalpha and HNF6 protein complex stimulated HNF6-dependent transcriptional activity through recruitment of the CBP co-activator protein in HepG2 cells. There is evidence for C/EBPalpha to physically interact with HNF6 but HNF6 also targets the C/EBPalpha promoter to induce transcriptional activation.

In this regard it is of paramount importance that C/EBPalpha inhibited cell growth through direct repression of E2F mediated S-phase gene transcription. Specifically, the study of Slomiany et al. demonstrated the physical association of C/EBPalpha with S-phase gene promoters and its transcriptional repression [22]. Consequently, C/EBPalpha inhibited cell growth. It is also of considerable interest that the cyclin-dependend kinase 2 was highly significantly upregulated (p<0.01) in tissue extracts of colorectal liver metastases of male and female patients (see Lehner, Klempnauer and Borlak 2010, manuscript in preparation) and it was shown earlier that C/EBPalpha interacts directly with cdk2 to arrest cell proliferation through inhibition of this kinase. Thus, C/EBPalpha inhibited cdk2 activity by blocking the association of cdk2 with cyclins to bring about growth arrest.

Consequently, HNF6 links C/EBPα to cell cycle regulation. Here we show inhibition of FOXA2 to stimulate HNF6 activity, as evidenced by cell cycle analysis and BrdU cell proliferation assays, all of which demonstrates inhibition of growth, i.e. cell cycle arrest at the G1 and the G2/M phase (see Fig. 7). However, the role of FOXA2 in the regulation of HNF6 activity remains controversial. Some investigators suggest HNF6 to function as a coactivator protein to potentiate the transcriptional activity of FOXA2 [18], whereas others report HNF6 function to be independent of FOXA2 [20]. Here we show FOXA2 gene expression to be reduced in HNF6 plasmid expressing CaCo-2 cells (see Fig. 4). Previously it was shown that patients diagnosed with secondary liver malignancies display significant regulation of FOXA2 and HNF6 in nuclear extracts of colorectal liver metastases. We found HNF6 DNA binding to be selectively abrogated as a result of impaired HNF6 acetylation and interaction with FOXA2. In line with our previous clinical study and our recent study on the gene expression patterning of liver enriched transcription factors in different segments of the human intestine we now report the HNF6 protein to be below the level of detection in Caco-2 cell cultures, even though expression of HNF6 mRNA could be evidenced, but was approximately half of that observed for FOXA2 (see Fig. 2C). Note, expression of HNF6 protein expression in Caco-2 and HepG2 cells is below the level of detection. It is of considerable importance that siRNA-mediated functional knockdown of FOXA2 resulted in transcriptional activation of HNF6 and of genes targeted by this factor. Our findings with the human colon cancer cell line Caco-2 agreed well with previous studies on the human hepatoma HepG2 cell line co-transfected with HNF6 or its deletion mutants as well as FOXA1, FOXA2, or FOXA3 TATA-luciferase reporter constructs [18]. In the present study we transfected HNF6 into HepG2 cells. This resulted in cell cycle arrest in the G1 phase. Likewise, cell proliferation was significantly reduced in the BrdU labeling assay, therefore confirming an important inhibitory role of HNF6 in the regulation of cell cycle progression and cell proliferation of cancer cells. Overall, results from the human Caco-2 and HepG2 cells agreed well. Importantly, FOXA2 protein was strongly induced in human colorectal liver metastases [3] and the findings of the present study are highly suggestive for an inhibitory crosstalk of FOXA2 and HNF6 in colorectal liver metastases. There is a report to suggest HNF6 activity to be independent of FOXA2 [20] and in this conditional FOXA2 knockout mouse model targeted expression of HNF6 genes appeared to be independent of the presence of FOXA2. In our previous clinical study, however, HNF6 was not expressed in healthy or cancerous colon, but was abundantly expressed in nuclear extracts of colorectal liver metastatic tissue. Nonetheless, HNF6 DNA binding activity was selectively abrogated in colorectal liver metastases because the protein was not acetylated and this posttranslational modification is a prerequisite for DNA binding activity at targeted promoters. As shown in the present study HNF6 is detrimental to malignantly transformed cells and blocks cell cycle progression. Further evidence stems from siRNA-mediated functional knockdown of FOXA2, which recovered HNF6 activity and caused cell cycle arrest.

Overall, HNF6 stimulated C/EBPα-dependent transcription [14] and resulted in an approximately 6-fold increased in C/EBPα gene expression in transfected Caco-2 cell cultures. A functional link between recovery of HNF6 activity and C/EBPα dependent cell cycle regulation was established by cell cycle analysis and BrdU labeling assay. The fact that HNF6 gene expression was increased as a result of FOXA2 siRNA-mediated functional knockdown provides further evidence for an inhibitory crosstalk between FOXA2 and HNF6 as there is conclusive evidence for C/EBPα to bring about growth arrest by inhibiting Cdk2 and Cdk4 [21].

In conclusion, siRNA mediated functional knockdown of FOXA2 increased transcriptional activity of HNF6 and of genes targeted by this factor. Recovery of HNF6 activity resulted in cell cycle arrest in human tumor gut epithelium and liver parenchyma cancer cell lines. Our study demonstrates a significant role of FOXA2 in colorectal liver metastases, which makes FOXA2 an interesting target in the therapy of colorectal liver metastases.
